# Impact of dialysis dependence on prognosis in patients with myocardial infarction: An 11-year population-based study

**DOI:** 10.1097/MD.0000000000009833

**Published:** 2018-02-09

**Authors:** Chung-Ming Fu, Chih-Hsiang Chang, Cheng-Chia Lee, Pei-Chun Fan, Shao-Wei Chen, Chien-Te Lee, Chien-Hsing Wu, Lung-Chih Li, Tien-Hsing Chen

**Affiliations:** a Division of Nephrology, Department of Internal Medicine, Kaohsiung Chang Gung Memorial Hospital and Chang Gung University College of Medicine, Kaohsiung; b Department of Nephrology, Kidney Research Center, Chang Gung Memorial Hospital, Linkou Medical Center, Chang Gung University; c Department of Cardiothoracic and Vascular Surgery, Chang Gung Memorial Hospital, Linkou Medical Center, Taoyuan; d Division of Cardiology, Department of Internal Medicine, Chang Gung Memorial Hospital, Keelung, Taiwan.

**Keywords:** acute myocardial infarction, chronic kidney disease, coronary artery disease, dialysis, prognosis

## Abstract

In this study we aimed to directly compare the short and long-term prognosis of nondialysis patients with chronic kidney disease (CKD), dialysis patients, and patients with preserved renal function after acute myocardial infarction (AMI).

AMI in patients with CKD is a catastrophic event associated with high medical expenditures and dismal survival. However, there is little research comparing post-AMI outcomes between patients with CKD who were and were not receiving dialysis.

The retrospective cohort study included patients with AMI (n = 158,125) in the Taiwan's National Health Insurance Research Database who were treated March 1998 and December 2009. Patients were classified into a nondialysis CKD group (n = 6300), dialysis group (n = 5140), and a control group (n = 146,685). The clinical characteristics, in-hospital events, and long-term outcomes of these 3 groups were compared separately using a multivariable Cox proportional hazard model.

The risks of in-hospital death and 2-year all-cause mortality were the highest in the dialysis group, followed by the nondialysis CKD group, and were the lowest in the control group. The 1-year risk of myocardial infarction did not differ among the 3 study groups, but the 2-year risk of myocardial infarction was higher in the dialysis group than in the control group (hazard ratio, 1.13; 95% confidence interval, 1.03–1.24; *P* = .010).

Patients with CKD experienced adverse short- and long-term outcomes after acute myocardial infarction. Patients with CKD, especially those who are dialysis dependent, may require more intensive management to improve their post-AMI clinical outcomes.

## Abstract 2

Supplemental Digital Content is available in the text

## 1 Introduction

Chronic kidney disease (CKD) is a global health problem associated with poor cardiovascular outcomes,^[1–3]^ and the population of patients with CKD and end-stage renal disease (ESRD) is increasing worldwide.^[1]^ The United States Renal Data System (USRDS) reported that there were 661,648 patients with ESRD in the United States (2034 per million) in 2013, and the prevalence of treated ESRD in Taiwan and in Japan was even higher. The Medicare expenditures for ESRD in United States in 2013 totaled 30.9 billion dollars. Most of these expenditures were attributed to inpatient hospital admissions.^[1,4]^

Acute myocardial infarction (AMI) in patients with CKD is a catastrophic event associated with high in-hospital expenses and dismal survival rates.^[5–8]^ Data reported from the USRDS found that the 2-year mortality rate of patients with AMI with the diagnosis of CKD in the United States between 2011 and 2013 was 61%, compared to 43% for patient without CKD.^[1]^ Prior studies have reported that patients with AMI with CKD or dialysis-dependent status were less likely to receive adequate coronary artery interventions and cardioprotective medications compared to patients with preserved renal function.^[5,6,9,10]^ The lower implementation of the standard of care in these patients may be due to several factors, including concerns about contrast-induced nephropathy, or the possible poor prognosis in patients with multiple comorbidities. Moreover, data on the long-term outcomes of nondialysis CKD and dialysis patients following AMI are sparse.^[9,11–14]^

Taiwan has one of the highest prevalence rates of ESRD.^[1]^ In this population-based study, we used the National Health Insurance Research Database (NHIRD) in Taiwan to monitor 158,125 patients with AMI with preserved renal function, nondialysis CKD, and dialysis-dependent CKD, to determine their rates of short- and long-term recurrent myocardial infarction (MI), gastrointestinal bleeding, all-cause mortality, and cardiovascular death.

## 2 Methods

### 2.1 Data source

In this population-based cohort study, the data were analyzed retrospectively from the NHIRD, which includes detailed health-care data from the universal demographic and enrollment records, hospital admissions, outpatient visits, disease profiles, prescriptions, and interventional procedures. The NHIRD is maintained by the National Health Insurance (NHI) program that comprehensively covers the medical needs of 99.19% of the population in Taiwan, a group of more than 23 million people. All of the diagnoses in this database were assigned utilizing the International Classification of Disease-9 (ICD-9) codes. Since all of the personal information and original identification numbers were encrypted before our analysis, no informed consent was required. This study was approved by the Ethics Institutional Review Board of Chang Gung Memorial Hospital (103-6077B).

### 2.2 Study patients and design

**Figure 1:**
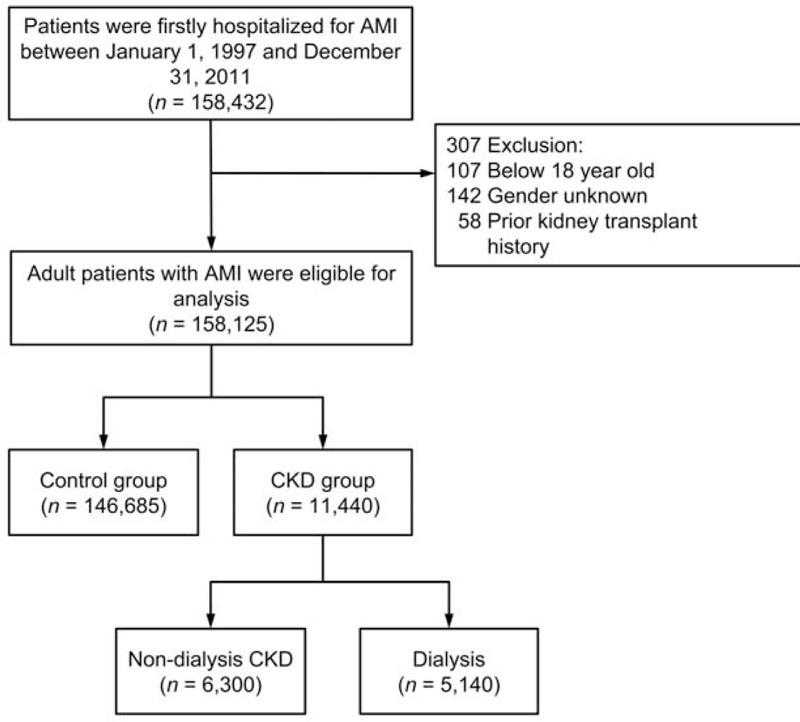
Flowchart of inclusion. After relevant exclusions, patients hospitalized with a diagnosis of AMI were included in our analysis and classified into a nondialysis CKD group, dialysis group, and a control group. AMI = acute myocardial infarction, CKD = chronic kidney disease.

We identified all patients in the NHIRD who were firstly admitted for an AMI (ICD-9-CM codes-410) between March 1, 1998 and December 31, 2009. These patients were classified into CKD and control group. The diagnosis of CKD was based on the ICD-9 CM code 585 assigned during their hospitalization. The CKD group was further classified into the nondialysis CKD and dialysis subgroups. The dialysis status was determined by both the specific ICD-9-CM codes and the patients’ registration in the Registry for Catastrophic Illness Patient Database, a subsection of the NHIRD. In Taiwan, patients can be registered for kidney failure only if: (1) they are receiving maintenance dialysis therapy with a permanent dialysis route such as arteriovenous fistula or tunneled cuffed catheter in HD patients, or a peritoneal dialysis catheter in PD dialysis patients; (2) their estimated glomerular filtration rate (eGFR) is <10 mL/min/1.73 m^2^ in nondiabetes mellitus (DM) patients or eGFR <15 mL/min/1.73 m^2^ in patients with DM; and (3) they have evidence of chronic renal parenchymal change on renal ultrasonography or a documented history of CKD for more than 3 months. The validity of NHIRD data and the accuracy of the diagnoses of major diseases such as MI, CKD, and dialysis status, have been described in previous studies.^[15–19]^ Patients aged <18 years were excluded. In addition, patients with a history of kidney transplant before admission were also excluded. The index hospitalization was the date when the patient was admitted for an AMI. The follow-up period was defined as the time from the index hospitalization to December 31, 2009, or whenever the patients died or were lost to follow-up. The enrollment flow chart is shown in Figure 1.

### 2.3 Outcome measures

Information on the patients’ baseline characteristics, comorbidities, medications, and in-hospital interventions including percutaneous coronary intervention (PCI) and coronary artery bypass grafting (CABG) were collected. The dialysis modalities of all the patients were also evaluated. The primary study outcomes were all-cause mortality and cardiac death at 2 years. Cardiovascular death was defined based on the criteria of the Standardized Definitions for End Point Events in Cardiovascular Trials published by the Food and Drug Administration.^[20]^ The date and cause of death were obtained from the NHIRD registry data. The other study endpoints include recurrent MI (ICD-9 code: 410) and gastrointestinal bleeding (ICD-9 code: 578).

### 2.4 Statistical analysis

We compared the patient characteristics among the study groups using the 1-way analysis of variance for continuous variables and the chi-square test for categorical variables. The risk of in-hospital outcomes among the study groups was compared using multivariable logistic regression for categorical outcomes and multivariable linear regression for continuous outcomes (ie, length of hospital stay). The time to event data (follow-up outcomes) among the study groups was compared using a multivariable Cox proportional hazard model. The patients’ characteristics listed in Table 1 were adjusted in the multivariable analyses, except for the follow-up year. We performed the data analyses using SPSS 22 (IBM SPSS, IBM Corp, Armonk, NY).

**Table 1:**
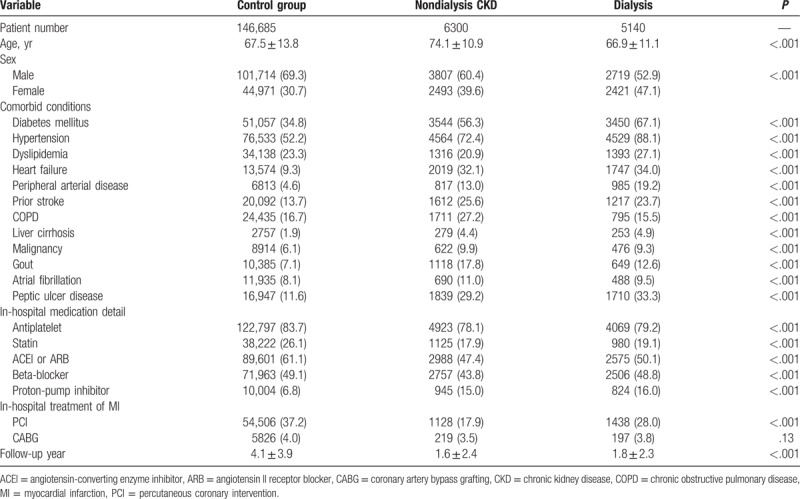
Baseline characteristics of patients (N = 158,125).

## 3 Results

### 3.1 Patient's characteristics

This study included 158,125 patients; 6300 (4.0%) of the patients had CKD but were not receiving dialysis (the nondialysis CKD group), and 5140 (3.3%) patients were dependent on renal replacement therapy (the dialysis group). Table 1 lists the patient's baseline characteristics. The nondialysis patients with CKD were the oldest among the study groups (74.1 ± 10.9 years). Generally, the prevalence of comorbid diseases in the 2 CKD groups (nondialysis CKD and dialysis) was higher than in the control group. During the index admission, the patients in the control group were more likely to be prescribed antiplatelet therapy, statins, angiotensin-converting enzyme inhibitors or angiotensin II receptor blockers, and beta-blockers compared to the patients in either of the 2 CKD groups; the phenomenon was reversed in the case of proton-pump inhibitors. Patients in the control group were most likely to undergo PCI (37.2%), followed by the dialysis group (28.0%) and then the nondialysis CKD group (17.9%). The mean duration of dialysis for the patients in the dialysis group was 3.5 ± 3.0 years and hemodialysis was the predominant dialysis modality (92.6%) (data not shown).

### 3.2 In-hospital event and outcome

Table 2 displays the in-hospital event/outcomes comparison across the study groups. The use of mechanical circulatory support (intra-aortic balloon pump or extracorporeal membrane oxygenation) was similar among the study groups. After adjusting for patient characteristics, the risks of cardiogenic shock and in-hospital death were the highest in the dialysis group, followed by the nondialysis CKD group, and the lowest in the control group. The risk of in-hospital gastrointestinal bleeding was higher in the dialysis group than in the other 2 groups; however, no difference was found between the nondialysis CKD and control groups. The length of intensive care unit (ICU) stay and length of hospital stay were the longest in the nondialysis CKD group, followed by the dialysis group, and they were the shortest in the control group.

**Table 2:**
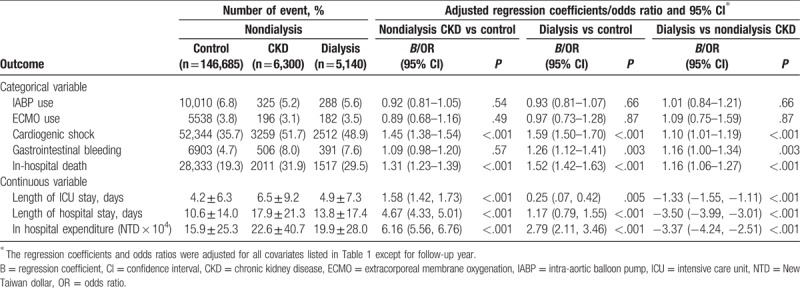
In-hospital event and outcome (N = 158,125).

### 3.3 Follow-up outcomes

**Figure 2:**
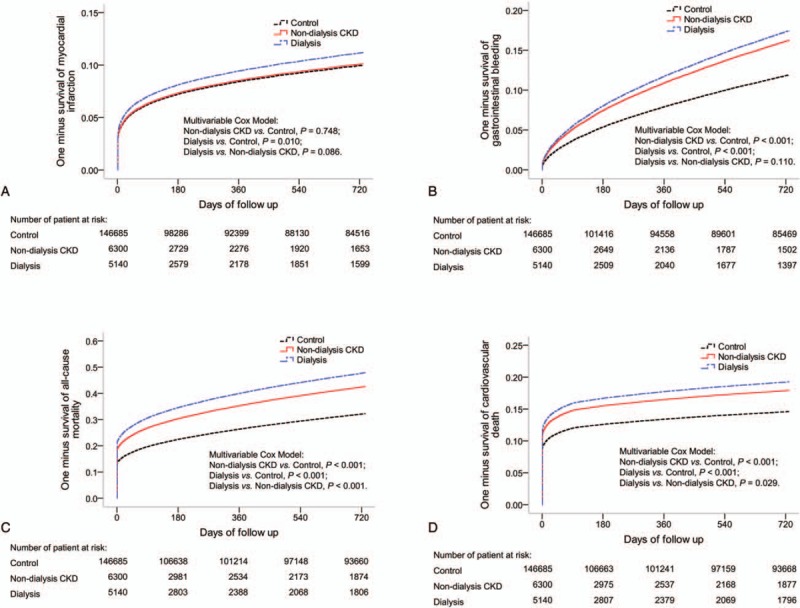
Adjusted survival curves of myocardial infarction (A), gastrointestinal bleeding (B), all-cause mortality (C), and cardiovascular death (D) during the 2-year follow-up period.

Table 3 summarizes the follow-up outcomes comparison across the study groups. The 1-year risk of recurrent myocardial infarction did not differ among the 3 groups; however, the 2-year risk of myocardial infarction was higher in the dialysis group than in the control group [hazard ratio (HR), 1.13; 95% confidence interval (CI), 1.03–1.24]. Patients in the 2 CKD groups had a higher risk of gastrointestinal bleeding at both the 1- and 2-year follow-ups compared to the patients in the control group, but the risk was comparable between the 2 CKD groups. During the 1- and 2-year follow-ups, the risk of all-cause mortality was the greatest in the dialysis group, and the risk in the nondialysis CKD group was greater than that in the control group. The risk of CV death in the 2 CKD groups was greater than that in the control group, but the risk was slightly higher in the dialysis group than in the nondialysis CKD group at the 1-year (HR, 1.07; 95% CI, 1.00–1.16; *P* = .063) and 2-year follow-ups (HR, 1.08; 95% CI, 1.01–1.16; *P* = .029). Figure 2 depicts the adjusted survival curves of the study groups during the 2-year follow-up period.

**Table 3:**
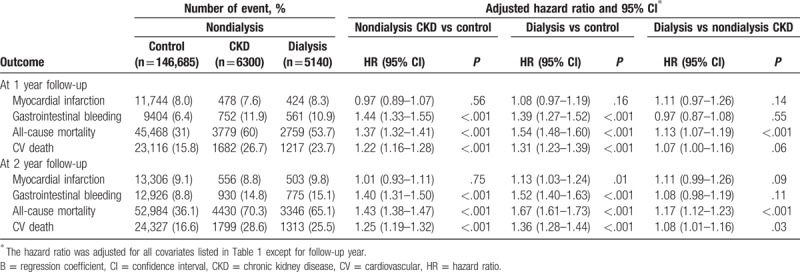
Primary outcomes in various follow-up periods (N = 158,125).

## 4 Discussion

In this study, we determined that after AMI, patients with CKD experienced poorer in-hospital and long-term fatal outcomes, whether they were dialysis dependent or not. The reasons for the poorer outcomes among the patients with CKD might be due to multiple factors. Patients with CKD are typically older and have more comorbidities.^[21,22]^ In addition, it can be more difficult to recognize an early AMI in patients with CKD, because they are less likely to have typical symptoms and electrocardiography findings during an AMI.^[11,23]^ Observational studies have demonstrated that renal insufficiency is not only an important risk factor for coronary artery disease, but also a predictor of poorer outcome in patients after myocardial infarction.^[3,8,21,24–28]^ The possible explanations might include endothelial dysfunction and atherosclerosis secondary to the accumulation of uremic toxins.^[29–31]^ Patients with kidney failure are also more likely to have vascular calcification, which can contribute to poor cardiovascular outcomes.^[32,33]^

Patients with renal insufficiency were frequently excluded from prospective cardiovascular trials, and few trials have directly compared outcomes in nondialysis patients with CKD and dialysis patients. A meta-analysis from Bundhun et al^[34]^ analyzed the impact between PCI and CABG, in patients with CKD and those on chronic dialysis, PCI associated with significantly higher mortality during a long-term follow-up period but no substantially different in short-term outcome. In our study, we independently compared the short- and long-term outcomes of nondialysis patients with CKD and dialysis patients. Our results demonstrated that the dialysis patients had worse in-hospital outcomes including death, GI bleeding, and cardiogenic shock, compared to the nondialysis patients with CKD. The dialysis patients also had a higher rate of all-cause mortality at 2 years. The risk of CV death was also slightly higher in the dialysis group.

Compared to the dialysis patients, the nondialysis patients with CKD had longer ICU stays, longer hospital stays, and higher hospital expenses. We wonder that one major reason is higher in-hospital mortality rate of dialysis patients.^[12,23,28,35]^ Some medical providers might have withheld cardiac catheterization due to concerns about contrast nephrotoxicity, or they may have made more effort to prevent or to manage contrast-induced nephropathy before and after cardiac catheterization. These management considerations might have added to the cost and length of the nondialysis CKD patients’ hospitalizations. Previous studies have confirmed that the use of thrombolysis, PCI, and other acute cardiac interventions were less common in patients with advanced kidney disease and dialysis.^[5,6,9,10]^ Medical providers may also be hesitant to provide aggressive treatment due to the poor expected outcome of patients with advanced renal disease. Bae et al^[36]^ reported that patients with lower GFR receive less aggressive treatments and are less likely to undergo revascularization after AMI compared to control patients.

It was well known that patients with CKD have a higher risk of developing gastrointestinal bleeding than patients with preserved renal function have,^[37–39]^ but few studies have done a head-to-head comparison between nondialysis CKD and dialysis patients on this issue. A collaborative research project of the USRDS and Third National Registry of Myocardial infarction^[11]^ reported that the adjusted likelihood of in-hospital major bleeding of advanced CKD patients (nondialysis) was higher than that of dialysis patients. Another trial by Nikolsky et al^[12]^ investigated 1575 patients with DM who underwent PCI, and showed that nondialysis CKD and dialysis patients had a higher risk of in-hospital GI bleeding compared to patients without CKD. In our study, the dialysis group had a higher risk of in-hospital GI bleeding compared to the other 2 groups. However, there was no difference between nondialysis and control group. The reasons for the discrepancy between our results on in-hospital GI bleeding and the results of prior studies were unclear. In our study, the nondialysis patients with CKD were the oldest of the 3 groups, and the results from the prior studies were not corrected for age and other multiple variables. The higher rates of in-hospital GI bleeding in the dialysis group have resulted from platelet dysfunction due to severe renal insufficiency, heparinization during hemodialysis, or the occurrence of hemorrhagic ulceration after cardiogenic shock.^[38,40]^ In patients with less severe kidney disease, it may be that age and other comorbidities, but not renal insufficiency itself, contribute to in-hospital GI bleeding after MI. This hypothesis needs to be confirmed in future studies.

There is little reported data on the long-term risk of gastrointestinal bleeding in patients with CKD after MI. In our study, up to 14.8% of the nondialysis patients with CKD and 15.1% of the dialysis patients experienced gastrointestinal bleeding during the 2-year follow-up period, compared with 8.8% in the control group. Platelet dysfunction due to uremia, progression of renal dysfunction in the CKD patients during the long-term follow-up, and excessive dosing of anticoagulopathy therapies may have contributed to this phenomenon.^[41]^

It is noteworthy that in our study the risk of recurrent MI in the nondialysis patients with CKD did not significantly differ from the risk of recurrent MI in the control patients at 1 and 2 years. The rates of recurrent MI between the dialysis and nondialysis patients at 1 and 2 years were also not significantly different. These findings are different from several previous studies^[5,8,24]^ but are consistent with 1 recent Korea registry study, in which patients with CKD had an increased risk of cardiovascular death at 1 year but did not have a concomitant increased risk of MI.^[36]^ We proposed several hypotheses to explain this discrepancy: the use of guideline-recommended antiplatelet agents for patients with CKD after MI was prevalent in recent years, especially in Taiwan under the comprehensive support of national health insurance; the more prevalent usage of drug-eluting stents; improvements in post-MI clinical care; and increased awareness about CKD. These hypotheses might explain the improved outcomes of recurrent MI in patients with CKD, but they require confirmation in future studies.

The study had several limitations. First, this was a retrospective registry study, and the association between the patients’ characteristics and their clinical outcomes may not be causal. However, large cohort observational studies can be informative. Second, the NHIRD database is inherently limited for CKD studies because it does not include data on creatinine levels and eGFR. Thus, information on CKD stages could not be provided in this study. However, the accuracy of the CKD diagnosis in the NHIRD has been validated by previous studies.^[15–17]^ The definition of dialysis patients in our studies was also rigorously designed. The duration of CKD and dialysis might affect the outcome. Further investigation about the duration of CKD might be needed to clear the association between CKD duration and AMI. Finally, although we did have access to information about the medications the patients received during their hospitalizations, information on their medication use during follow-up period was not available.

## 5 Conclusion

We determined that patients with CKD had adverse short- and long-term outcomes after AMI. CKD and dialysis patients were less likely to receive evidence-based cardioprotective medications during their hospitalization, and had more episodes of gastrointestinal bleeding during follow-up after experiencing an AMI. From this head-to-head comparison study, we learned that the dialysis patients had even worse in-hospital and long-term outcomes compared to nondialysis patients with CKD, although there was no significant difference in the rate of recurrent myocardial infarction. Dialysis patients may require more intensive management to improve their post-AMI clinical outcomes.

## Acknowledgments

The study data were obtained from the NHIRD provided by the National Health Insurance administration, Ministry of Health and Welfare of Taiwan, and managed by the National Health Research Institutes of Taiwan. However, the interpretation and conclusions contained in this study do not represent the opinions of the National Health Insurance Administration, the Ministry of Health and Welfare of Taiwan, or the National Health Research Institutes of Taiwan. The authors thank Alfred Hsing-Fen Lin's for his assistance with the statistical analysis.

## Supplementary Material

Supplemental Digital Content
