# Supplementary material for: Impact of dialysis dependence on prognosis in patients with myocardial infarction: An 11-year population-based study
Source: Medicine (Baltimore). 2018 Feb 9;97(6):e9833. doi: 10.1097/MD.0000000000009833 (PMC5944684; doi:10.1097/MD.0000000000009833)
Supplement: Supplemental Digital Content [file medi-97-e9833-s001.docx]

**Supplementary Material**

Appendix. ICD-9-CM code used for diagnosis in the current study

| Variable | Code |
| --- | --- |
| Acute myocardial infarction | 410.xx |
| Chronic kidney disease | 585.xx |
| Dialysis | 585 (Catastrophic Illness Card) |
| Diabetes mellitus | 250.xx |
| Hypertension | 401.xx–405.xx |
| Dyslipidemia | 272.xx |
| Heart failure | 428.xx |
| Peripheral arterial disease | 440.0, 440.2x, 440.3x, 440.4, 440.9, 443.9, 444.2, 444.22, 444.8, 444.81, 445.0, 445.02, 250.7x, 707.1x |
| Stroke | 430.xx–437.xx |
| Chronic obstructive pulmonary disease | 490.xx–496.xx |
| Liver cirrhosis | 571.2, 571.5, 571.6 |
| Malignancy | 140.xx–208.xx |
| Gout | 274.xx |
| Atrial fibrillation | 427.31 |
| Peptic ulcer disease | 531.xx–534.xx |
| Gastrointestinal tract bleeding | 578.xx |
| Cardiovascular death |  |
| Acute myocardial infarction | 410.xx |
| Sudden cardiac death | 427.5 |
| Heart failure | 428.xx |
| Cardiogenic shock | 78551 |
| Stroke | 430.xx–437.xx |
| Dysrhythmia | 427.xx |
| Pulmonary embolism | 415.1, 415.11, 415.19 |
| Aortic aneurysm dissection | 441.xx |
| Ischemic heart disease | 411.xx–414.xx |
